# All Eyes on Al!

**DOI:** 10.4103/0974-7753.66924

**Published:** 2010

**Authors:** Patrick Yesudian

**Affiliations:** President, The Hair Research Society of India, Chennai, India

**Figure 1:**
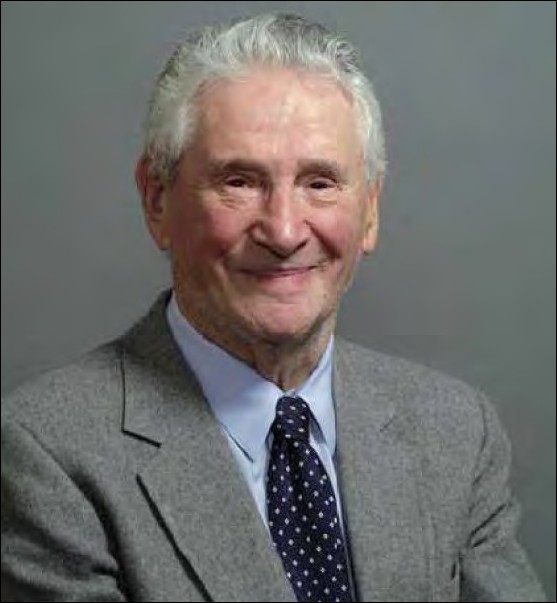
Albert Kligman

I had the privilege and opportunity of meeting Dr. Albert Kligman [Figure 1] in October 1995, at the launching of the new retinoid molecule, adapalene. Over 100 dermatologists were invited from all over the world for the Differin Symposium which was held at the Hotel Casino Royale in Nice, France. From India, late Dr. Ranjit Panja and I had been kindly invited by the organizers. The inaugural address was of course by none other that Dr. Kligman. He spoke for nearly an hour on acne and the mode of action of the new molecule adapalene and at the end of the talk, there were questions from the audience, which went on for another hour. I gathered many useful points from his talk, which I find useful in my practise even today. A few of his original observations of his were that the pigmented skins were prone for severe acne than the Caucasians and children who developed acne even before menarche should be treated aggressively with the topical tretinoin. He also said that he did not believe in cosmetic acne and that these were all stress induced. Though his views may not be universally accepted, I still preserve the notes I jotted down during his lecture.

At the banquet dinner that evening I had the good fortune of exchanging a few words with him. Though there were other international dermatological celebrities like Jablonska, Cunliffe, falabella, Leyden and Goh, Dr. Albert Kligman, fondly addressed by everyone as AL, was the cynosure of all eyes.

That was the first and the last time I saw AL Kligman but I shall forever remember him. Though he has departed from this world, as the hymn goes, “he will be remembered by what he has done.”

May his soul rest in peace!

